# 25-hydroxyvitamin D3 inhibits oxidative stress and ferroptosis in retinal microvascular endothelial cells induced by high glucose through down-regulation of miR-93

**DOI:** 10.1186/s12886-022-02762-8

**Published:** 2023-01-13

**Authors:** Dongmei Zhan, Juan Zhao, Qin Shi, Juan Lou, Weiling Wang

**Affiliations:** Department of Ophthalmology, General Hospital of Ningxia Medical University, Ningxia Hui Autonomous Region, 750004 Yinchuan, China

**Keywords:** Human retinal microvascular endothelial cells (hRMVECs), Oxidative stress, Ferroptosis, 25-hydroxyvitamin D3(25 (OH) D3), MiR-93

## Abstract

**Background:**

The decrease of vitamin D plays a critical role in diabetes mellitus (DM)-induced oxidative stress and vascular endothelial injury. Therefore, we investigated the effect and mechanism of 25-hydroxyvitamin D3 (25 (OH) D3) on oxidative stress and ferroptosis induced by high glucose in human retinal microvascular endothelial cells (hRMVECs). And the objective of this paper was to propose a new strategy for the prevention and treatment of diabetic retinopathy (DR).

**Methods:**

First, hRMVECs were transfected with mimics NC or miR-93. After that, cells were treated with 100 nM / 500 nM 25 (OH) D3 and then cultured in a high glucose (30 mM) environment. Subsequently, qRT-PCR was employed to detect the expression level of miR-93; CCK-8 for the proliferation of cells in each group; biochemical tests for the level of intracellular reactive oxygen species (ROS), malondialdehyde (MDA), reduced glutathione (GSH) and ferrous ion (Fe^2+^); and Western blot for the expression of ferroptosis-related proteins glutathione peroxidase 4 (GPX4) and SLC7A11).

**Results:**

Under a high glucose environment, 25 (OH) D3 at 100 nM/500 nM could significantly promote the proliferation of hRMVECs, remarkably decrease the level of intracellular ROS/MDA, and up-regulate the level of GSH. Besides, 25 (OH) D3 greatly reduced Fe^2+^ level in the cells while increased protein level of GPX4 and SLC7A11. Subsequently, we found that high glucose induced miR-93 expression, while 25 (OH) D3 markedly decreased high glucose-induced miR-93 overexpression. Furthermore, overexpression of miR-93 inhibited the functions of 25 (OH) D3 by activating ROS (ROS and MDA were up-regulated while GSH was down-regulated) and inducing Fe^2+^ (Fe^2+^ level was up-regulated while GPX4 and SLC7A11 level was down-regulated) in cells.

**Conclusion:**

25 (OH) D3 may inhibit oxidative stress and ferroptosis in hRMVECs induced by high glucose via down-regulation of miR-93.

**Supplementary Information:**

The online version contains supplementary material available at 10.1186/s12886-022-02762-8.

## Background

Diabetic retinopathy (DR) is not only a common microvascular complication of diabetes mellitus (DM) but also the main cause of blindness and visual loss in the working age group [1]. According to the International Diabetes Federation report, about 1/3 of the 425 million patients with DM worldwide are affected by DR [2]. In clinical practice, the treatment for DR is principally focused on prevention, including systematic management and control of the hyperglycemia, hypertension and hyperlipidemia in patients as well as vision protection by reducing vascular leakage [3]. However, current treatments are not satisfactory because they can only reduce the downstream outcomes of DR and are limited by the side effects [4–6]. Therefore, new strategies are needed to address the threat to human health posed by DR.

MicroRNA (miRNA) is a novel non-coding small RNA with about 22 nucleotides in length, which plays a key role in the pathogenesis of DM [7]. MiR-93, a newly discovered miRNA, exerts important functions in various diseases or injuries [8–13]. Several reports have revealed that miR-93 is associated with DR damage. For example, Hirota et al. observed evidently increased level of miR-93 in patients with proliferative DR [14], and a clinical study by Zou et al. also indicated that high plasma level of miR-93 was linked to a high risk of developing type 2 DR [15]. Subsequently, Ahmed et al. discovered that highly expressed miR-93 could induce inflammatory and oxidative stress responses in retinal pigment epithelium and promote the development of DR, suggesting the vital role of miR-93 in DR development [16].

Vitamin D (Vit D) is an important vitamin in the human body, and 25-hydroxyvitamin D3 (25 (OH) D3) is the main active substance of Vit D. Recently, many research has shown that Vit D also participates in cell proliferation and differentiation, and has immunomodulatory and regulatory properties [17]. A study by Codo Er-Franch et al. demonstrated notably lowered 25 (OH) D3 level in obese children with increased markers of oxidative stress, inflammation, and endothelial activation [18]. A clinical trial by Anandabaskar et al. revealed that continuous 8-week oral 25 (OH) D3 could improve vascular function and reduce oxidative stress in VD-deficient type 2 DM patients [19]. From the above, 25 (OH) D3 supplementation may be an effective strategy to alleviate DM-induced oxidative stress and angiogenesis. Nevertheless, there are no reports displaying the relationship between 25 (OH) D3 and DR currently. It’s reported that Vit D can regulate the transcription of miRNA genes through Vit D receptor binding to its sequence motif located in the promoter of target miRNA genes, miRNA maturation through regulating genes involved in miRNA processing or miRNA stability [20, 21]. Therefore, miRNAs could serve as biomarkers and molecular targets of Vit D which could be modulated by nutritional interventions in health and disease. In this study, we explored the effects of Vit D targets miR-93 on high glucose induction on the activity, oxidative stress and ferroptosis of human retinal microvascular endothelial cells (hRMVECs) in vitro.

## Materials and methods

### Cell culture and grouping treatment

HRMVECs were obtained from Cell Systems (Kirkland, USA) and cultured in a DMEM containing 10% fetal bovine serum (FBS, Gibco, USA) and 100 × penicillin streptomycin, and the cells were maintained in an incubator at 37 °C and 5% CO_2_.

Twenty-Five (OH) D3 was purchased from Shanghai Macklin Biochemical Technology Co., Ltd. And the grouping of hRMVECs was performed as follows. In the NG group and HG group, hRMVECs were treated at normal glucose (5 mM) and high glucose (30 mM) concentration for 48 h, respectively [22]. In 25 (OH) D3 100 nM and 25 (OH) D3 500 nM groups, hRMVECs were pretreated with 100 nM and 500 nM of 25 (OH) D3 for 3 h, respectively [23], and then the cells were induced by high glucose for 48 h.

Both miR-93 mimics and their control NC mimics were provided by Shanghai RiboBio Co., Ltd. According to the steps of lipo 2000 transfection, NC mimics or miR-93 mimics were transfected into hRMVECs. Afterwards, the cells were pretreated with 500 nM 25 (OH) D3 for 3 h, induced by high glucose for 48 h, and then were named as 25 (OH) D3 + NC group or 25 (OH) D3 + miR-93 group, respectively.

### qRT-PCR

Total RNA was extracted from cells using Trizol reagent (Sigma, USA), and then the quality and concentration of the extracted RNA were tested by standard denaturing agarose gel electrophoresis and NanoDrop spectrophotometer ND-8000. Next, RNA was reverse transcribed to synthesize cDNA based on the PrimeScript ™ RT Master Mix kit (Takara, Japan) instructions. Besides, miR-93 expression level was checked according to the instructions of SYBR Premix Ex TaqTM II kit (Takara, Japan). U6 served as an internal control. And the 2^−ΔΔCt^ method was adopted to calculate the relative expression of the target gene based on the experimental data obtained by qRT-PCR. The relevant primer sequences were displayed in Table 1.

**Table 1:** Quantitative Primer Sequences

Gene	Sequences (5’ to 3’)
miR-93	F: GCAGCAAACTTCTGAGACAC
	R: GTGCAGGGTCCGAGGTATTC
U6	F: CTCGCTTCGGCAGCACA
	R: AACGCTTCACGAATTTGCGT

### Cell viability detection

The treated hRMVECs were seeded in a 96-well plate at 5 × 10^3^ cells/well and detected after adherence at 0 and 24 h according to the instructions of CCK-8 reagent (Solarbio, China). To be specific, 10 µL CCK-8 reagent was added to each well, then the cells were incubated at 37 ℃ for 1–4 h. Later, the absorbance at 450 nm was detected utilizing a microplate reader and the proliferation rate was calculated.

### Biochemical tests

The treated hRMVECs were collected and the supernatant was removed. After being washed with PBS 3 times, the cells were collected using a cell scraper. Next, 2 mL PBS was added for the preparation of cell suspension. Subsequently, the suspension was centrifuged at 1000 × g at 4 ℃ for 10 min, and the supernatant was absorbed. Later, 300–500 µL homogenization medium was added and adequate mechanical crushing was performed. After that, the suspension was centrifuged at 12,000 r/min at 4 ℃ for 30 min, and the supernatant was placed on ice for testing. Finally, the level of ROS, malondialdehyde (MDA), reduced glutathione (GSH) and ferrous ion (Fe^2+^) in the cells of each group was measured by an automatic biochemical analyzer (Olympus, Japan) with the corresponding biochemical kits (Nanjing Jiancheng Bioengineering Institute, China).

### Western blot

Total cellular protein was extracted via RIPA lysate (Solarbio, China), and the concentration of extracted protein was determined with BCA kit (Beyotime, China). Later, 20 µg of treated proteins were separated by SDS-PAGE (sodium dodecyl sulfate polyacrylamide gel electrophoresis), and then the separated proteins were transferred to polyvinylidene fluoride (PVDF) membrane. After blocking with 5% non-fat dry milk for 1–3 h, the diluted primary antibodies (GPX4, ab125066; SLC7A11, ab175186; Abcam, UK) were added into the membrane for incubation at 4 ℃ overnight. The membrane was washed 3 times, and the secondary antibodies (bs-0295G-HRP, Bioworld, USA) were added for incubation at room temperature for 1 h. Afterwards, the membrane was washed 3 times again, and then electrogenerated chemiluminescence (ECL) reagent (Beyotime, China) was added. The proteins were developed and the image collection was performed in the gel imaging system; and the gray level of protein bands was analyzed using Image J software. Besides, GAPDH (ab9485) acted as an internal control to calculate the relative protein expression.

### Statistical analysis

The results were expressed as mean ± standard deviation (SD). SPSS 26.0 was employed to conduct one-way analysis of variance among multiple groups and independent samples T-test between two groups. And *P* < 0.05 was considered as a significant difference.

## Results

### 25-hydroxyvitamin D3 (25 (OH) D3) reduces the inhibitory effect of high glucose on the activity of human retinal microvascular endothelial cells (hRMVECs)

**Fig. 1:**
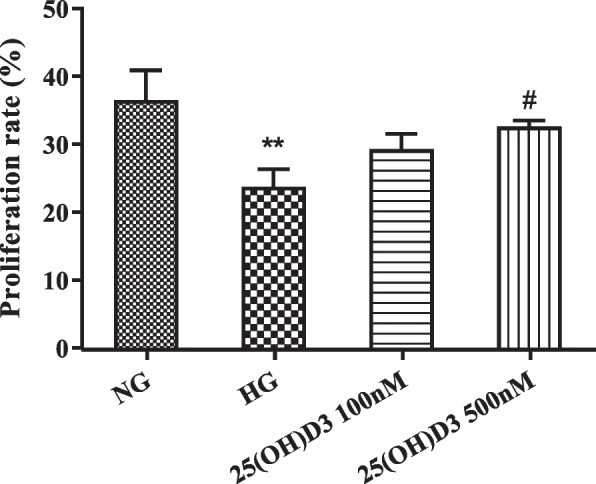
25-hydroxyvitamin D3 (25 (OH) D3) reduces the inhibitory effect of high glucose on the activity of human retinal microvascular endothelial cells (hRMVECs). The effect of different concentrations (100 nM and 500 nM) of 25 (OH) D3 on the cell viability of hRMVECs under high glucose environment determined by CCK-8, **P < 0.01 vs. NG group, #*P* < 0.05 vs. HG group

The examination outcomes presented that the cell proliferation rate in the HG group was much lower than that in the NG group (*P* < 0.01); compared with the HG group, the cell proliferation rates in the 25 (OH) D3 100 nM group and 25 (OH) D3 500 nM group were increased in a concentration-dependent manner (Fig. 1). The above indicated that high glucose could significantly inhibit the activity of hRMVECs, while 25 (OH) D3 could reduce the inhibitory effect of high glucose on the cell activity of hRMVECs.

### 25-hydroxyvitamin D3 alleviates the oxidative stress response induced by high glucose in human retinal microvascular endothelial cells

**Fig. 2:**
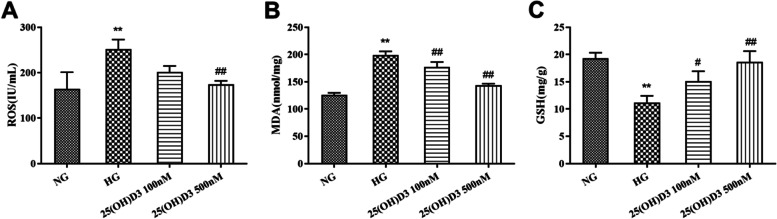
25-hydroxyvitamin D3 alleviates the oxidative stress response induced by high glucose in human retinal microvascular endothelial cells. **A**-**C**: Biochemical tests for the effect of different concentrations (100 nM and 500 nM) of 25 (OH) D3 on the level of oxidative stress substances (reactive oxygen species (ROS), malondialdehyde (MDA), reduced glutathione (GSH)) in hRMVECs induced by high glucose. **P < 0.01 vs. NG group, #*P* < 0.05 vs. HG group, ##*P* < 0.01 vs. HG group

Compared with the NG group, the HG group exhibited a noticeable increase in the ROS and MDA level while a marked decrease in the GSH level (Fig. 2 A-C, *P* < 0.01). Besides, compared with the HG group, the ROS and MDA level in 25 (OH) D3 100 nM group and 25 (OH) D3 500 nM group declined, while the GSH level was raised greatly in a concentration-dependent manner (Fig. 2 A-C, *P* < 0.05). From the above, 25 (OH) D3 could inhibit the oxidative stress response induced by high glucose in hRMVECs.

### 25-hydroxyvitamin D3 inhibits high glucose-induced ferroptosis in human retinal microvascular endothelial cells

**Fig. 3:**
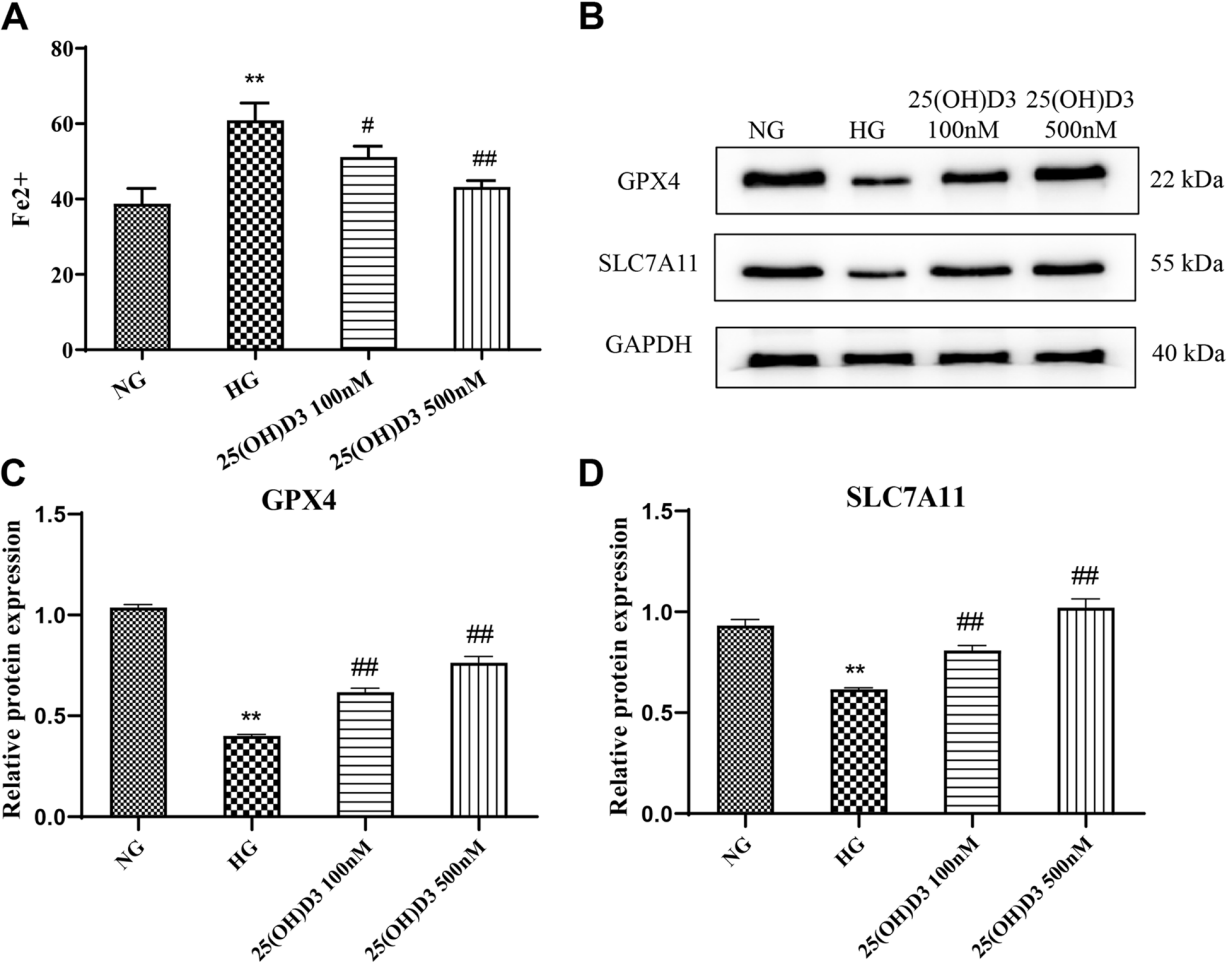
25-hydroxyvitamin D3 inhibits high glucose-induced ferroptosis in human retinal microvascular endothelial cells. A: Biochemical tests for the effect of different concentrations (100 nM and 500 nM) of 25 (OH) D3 on Fe^2+^ level in hRMVECs induced by high glucose; B/C: Western blot for detecting the effect of different concentrations (100 nM and 500 nM) of 25 (OH) D3 on the expression of ferroptosis-related proteins (GPX4 and SLC7A11) in hRMVECs induced by high glucose, **P < 0.01 vs. NG group, #*P* < 0.05 vs. HG group, ##*P* < 0.01 vs. HG group

The results revealed that the Fe^2+^ level in hRMVECs in the HG group was remarkably elevated compared with the NG group (*P* < 0.01). In addition, 25 (OH) D3 at 100 nM and 500 nM significantly inhibited the increase of intracellular Fe^2+^ level induced by high glucose in a concentration-dependent manner (Fig. 3 A, *P* < 0.05). In addition, Western blot results displayed an evident decrease in the protein level of GPX4 and SLC7A11 in the HG group in comparison with that in the NG group. Also, the protein level of GPX4 and SLC7A11 in the 25 (OH) D3 100 nM group and 25 (OH) D3 500 nM group was a lot higher than that in the HG group in a concentration-dependent manner (Fig. 3B-D, *P* < 0.01). All in all, high glucose induced ferroptosis in hRMVECs, and 25 (OH) D3 significantly inhibited ferroptosis in hRMVECs induced by high glucose.

### 25-hydroxyvitamin D3 can down-regulate miR-93 expression in human retinal microvascular endothelial cells induced by high glucose

**Fig. 4:**
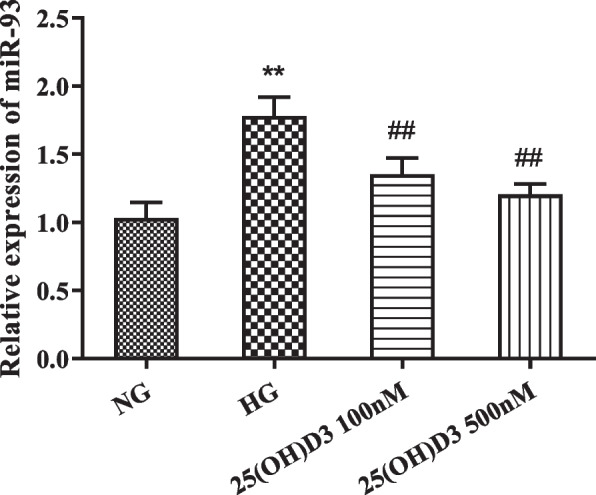
25-hydroxyvitamin D3 can down-regulate miR-93 expression in human retinal microvascular endothelial cells induced by high glucose. QRT-PCR was performed to detect the effect of different concentrations (100 nM and 500 nM) of 25 (OH) D3 on miR-93 expression in hRMVECs induced by high glucose, **P < 0.01 vs. NG group, ##*P* < 0.01 vs. HG group

The results manifested that miR-93 expression level in hRMVECs in the HG group was obviously increased compared with the NG group (*P* < 0.01); while pretreatment with 100 nM and 500 nM 25 (OH) D3 could evidently reduce miR-93 expression level in cells in a concentration-dependent manner (Fig. 4, *P* < 0.01).

### MiR-93 overexpression reverses the effect of 25-hydroxyvitamin D3 on oxidative stress and ferroptosis induced by high glucose in human retinal microvascular endothelial cells

**Fig. 5:**
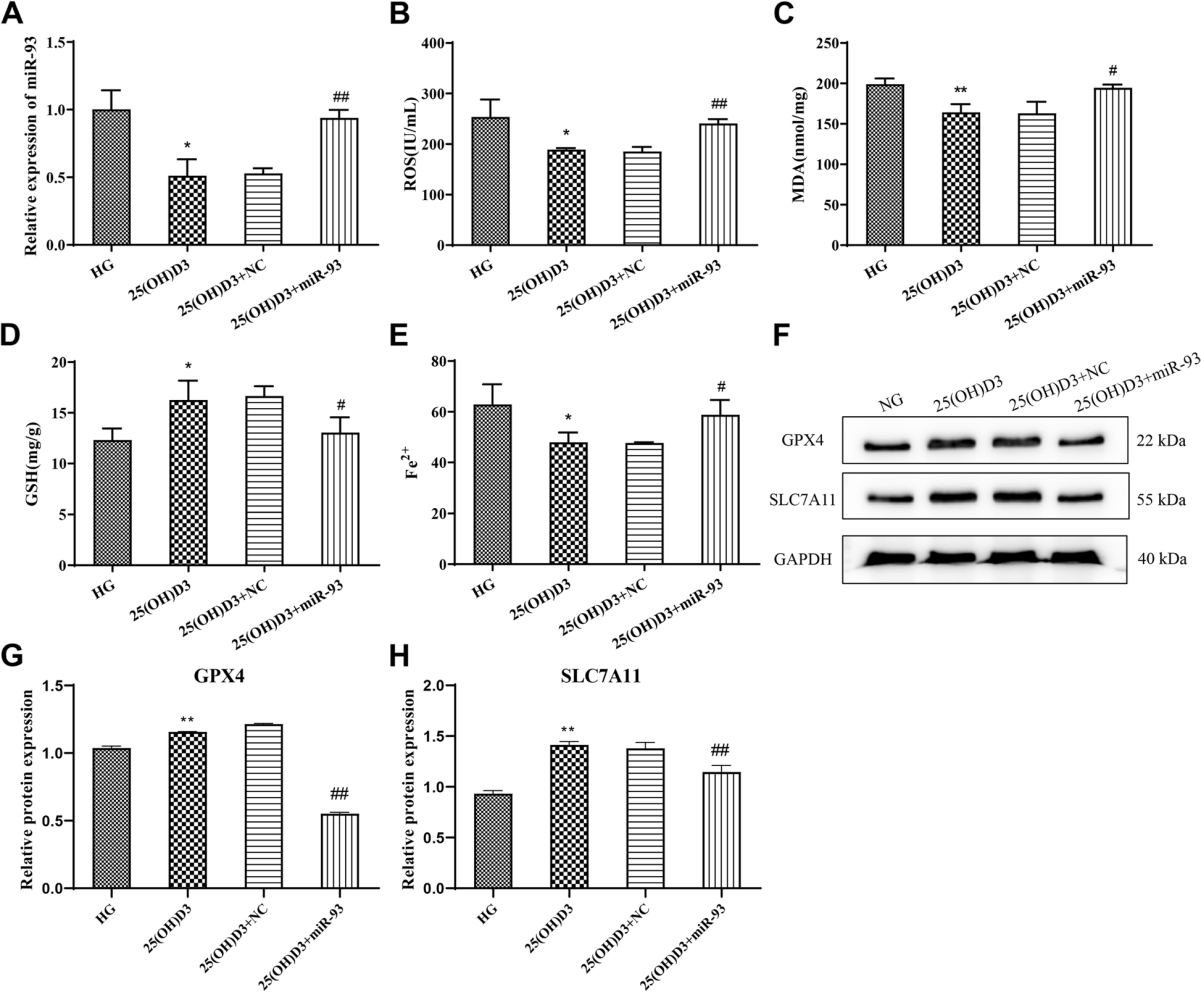
miR-93 overexpression reverses the effect of 25-hydroxyvitamin D3 on high glucose-induced oxidative stress and ferroptosis in human retinal microvascular endothelial cells. **A**: qRT-PCR for testing the expression of miR-93 in hRMVECs induced by high glucose in each group; **B**-**E**: Biochemical assay for detecting the level of ROS (**B**), MDA (**C**), GSH (**D**) and Fe^2+^ (**E**) in hRMVECs induced by high glucose in each group. **F**-**H**: Western blot for checking the protein expression level of ferroptosis-related proteins (GPX4 and SLC7A11) in hRMVECs induced by high glucose in each group, *P < 0.05 and **P < 0.01 vs. HG group, #*P* < 0.05 and ##*P* < 0.01 vs. 25 (OH) D3 + NC group

QRT-PCR results demonstrated that miR-93 was significantly down-regulated in cells treated with 25 (OH) D3, while miR-93 mimics transfection significantly reversed the inhibitory effect of 25 (OH) D3 on miR-93 expression in cells (Fig. 5 A, *P* < 0.01). Moreover, we examined the oxidative stress-related substances and ferroptosis-related parameters in each group of cells. And the examination outcomes showed that miR-93 mimics could reverse the inhibitory effect of 25 (OH) D3 on high glucose-induced oxidative stress (ROS and MDA level was up-regulated, while GSH level was down-regulated) (Fig. 5B-D, *P* < 0.05) and ferroptosis in hRMVECs (Fe^2+^ level and protein level of GPX4 and SLC7A11 was greatly lowered) (Fig. 5E-H, *P* < 0.05). In summary, miR-93 mimics could reverse the inhibitory effect of 25 (OH) D3 on high glucose-induced oxidative stress and ferroptosis in hRMVECs.

## Discussion

DR has become a worldwide concern, which can gradually develop into the proliferative vitreoretinopathy (PVR), leading to significant vision loss, and even blindness [24]. Therefore, early intervention and diagnosis can help delay the progression of the disease. The mechanism of DR is complex, including pericyte loss, neovascularization (NV), glycation end products, oxidative stress and ROS, inflammatory response and so on [25]. Currently, anti-vascular endothelial-derived growth factor (VEGF) therapy is widely used in clinical practice, carried out by means of different agents, including aflibercept (Eylea1), bevacizumab (Avastin1) and ranibizumab (Lucentis1) [26]. The VEGF family consists of six proteins: VEGF-A,-B,-C,-D,-E, and placental growth factor (PGF) [27]. Giurdanella et al. revealed that intravitreally injected anti-VEGF may exert relevant effect on retinal pericytes [28]. Usui-Ouchi et al. showed that intra-vitreal injection of the CITED2 peptide inhibited retinal NV by down-regulation of HIF-mediated transcription in retinal cells [29]. However, the adverse effects after the administration of high-dose anti-VEGF drugs can not be ignored [30]. It’s necessary to find alternative drugs without adverse effects to treat DR.

Recently, in vitro studies have shown that the active form of vit D has an effect of inhibiting neovascularization by HIF-1α/VEGF pathway [31, 32]. A large number of data have reported that Vit D level in the blood of DM patients is noticeably declined, and VD supplementation can greatly reduce insulin resistance in patients [33]. The study by Khumaedi et al. pointed out that the DM-induced ROS accumulation not only was the key to causing the oxidative response of retinaldehyde but also resulted in retinal vascular endothelial tissue damage and loss of endothelial cells and then promoted the process of DR [34]. In this study, 25 (OH) D3 remarkably alleviated the proliferation capacity reduction and oxidative stress state of hRMVECs caused by high glucose. Some previous research also have reported good antioxidant effect of Vit D. For instance, Tohari et al. stated that Vit D was able to protect retinal pigment epithelial cells from high glucose-induced oxidative damage and inflammatory damage through high glucose-treated ARPE-19 cells and streptozotocin-induced diabetic mouse models [35]. Zhu et al. claimed that Vit D could inhibit high glucose-induced oxidative stress in tubular cells via the AKT/UCP2 signaling pathway [36]. And it can be known from previous studies that the antioxidant functions of 25 (OH) D3 may be related to the reduction of inflammatory level [37].

Ferroptosis is a novel mode of non-apoptotic cell death, and its main features include intracellular iron overload, iron-dependent lipid peroxide accumulation, and oxidoreductase deficiency (especially GPX4) [38]. Several studies have displayed that ferroptosis is involved in DM pathogenesis. Additionally, ferroptosis also participates in the pathogenesis of diabetic complications such as myocardial ischemia and diabetic cardiomyopathy [39]. Many iron-containing proteins are involved in the phototransduction cascade in the retina [40]. Despite being an essential micronutrient for many protein functions, excessive irons are potentially harmful pro-oxidants [41]. Excessive irons result in the occurrence of Fenton reaction, catalyze the conversion of H_2_O_2_ to hydroxyl radicals, induce lipid peroxidation, DNA strand breaks and degradation of cellular components, and ultimately cause tissue damage [42]. As negative regulators of the ferroptosis, both GPX4 and SLC7A11 can greatly reduce the level of peroxidation in cells [43]. Also, we found that high glucose resulted in the accumulation of Fe^2+^ and the decrease of GPX4 and SLC7A11 protein level in hRMVECs, indicating that high glucose induced ferroptosis in hRMVECs. And our findings were consistent with the research of Singh and Zhu et al. that high glucose could induce ferroptosis in retinal pigment epithelial cells [44, 45]. Furthermore, we discovered that 25 (OH) D3 could reduce the high glucose-induced intracellular iron content, and notably increase the protein level of GPX4 and SLC7A11. The above results indicated that 25 (OH) D3 could effectively hinder the occurrence of ferroptosis induced by high glucose in hRMVECs. However, the specific mechanism of 25 (OH) D3 remains unknown and needs further exploration. There are no studies on the relationship between 25 (OH) D3 and ferroptosis, but 25 (OH) D3, another active component of Vit D, was found to be able to inhibit ferroptosis in zebrafish hepatocytes.

Liu et al. identified potential key genes of ferroptosis in the pathogenesis of intracerebral hemorrhage by bioinformatics analysis, and genes such as miR-93 and SNHG16 were screened and obtained in their report [46]. Related research has suggested that changes in the miR-93 level are evidently associated with high risk of DM [47]. And some studies manifested the correlation of the down-regulation of miR-93 expression with the imbalance of oxidative stress. For example, Su et al. revealed that up-regulation of miR-93 promoted oxidative stress and inflammatory response by activating RhoA/ROCK signaling pathway [48]. As demonstrated in a previous study, an increase of miR-93 expression is a leading cause of the occurrence of polycystic ovary syndrome (PCOS) and insulin resistance by targeting at GLUT4, an important protein in regulating glucose homeostasis [49]. Also, another experiment discovered that miR-93 expression is distinctly high in proliferative DR eyes with angiogenesis and proliferative responses, indicating that miR-93 may be associated with angiogenesis and fibrosis [50]. As mentioned above, miR-93 is a key signaling molecule that promotes the oxidative stress responses in cells and has been indicated as a significant regulator in DR. In addition, miR-93 is relevant to ferroptosis. In our study, high glucose could induce miR-93 expression in hRMVECs, while 25 (OH) D3 treatment effectively inhibited miR-93 expression in cells induced by high glucose; besides, overexpression of miR-93 could reverse the protective effect of 25 (OH) D3 on hRMVECs by promoting oxidative stress and ferroptosis. All the above indicated that 25 (OH) D3 inhibits the damages such as cell viability reduction, oxidative stress and ferroptosis in high glucose-induced hRMVECs. And the protective effects of 25 (OH) D3 on cells may be correlated with its down-regulation on miR-93 expression.

However, the function of vit D in DR is complex, it is a potent inhibitor of retinal neovascularization, and related to vessel related biomarkers [51]. In the process of DR, the epithelial-mesenchymal transition (EMT) develops in retinal pigment epithelium (RPE) cells, which transforms epithelial cells into stromal cells, enhancing their proliferation, migration and anti-apoptotic capacity, playing a key role in PVR formation [52, 53]. Bonventre and his colleagues reported the synthetic ligands of the vit D receptor can target the TGF-β-SMAD signaling pathway and inhibit the EMT progression [54]. Lai et al. showed that vit D can increase the protein expression of CD31, and reduce the protein expressions of alpha-smooth muscle actin (α-SMA) and fibronectin in the TGF-β1-induced fibrosis model. Additionally, the protein expression of VE-cadherin was increased and fibroblast-specific protein-1 (FSP1) was decreased after vit D treatment in the isoproterenol-induced fibrosis rat [55]. Vila et al. reported that active vit D rescued the uraemic medium-induced loss of cell-cell adhesion by increasing VE-cadherin and F-actin [56]. Collectively, Vit D is conducive to maintaining insulin secretion and improving insulin resistance, which can resist oxidative stress and ferroptosis, inhibit the growth of vascular smooth muscle cells, inhibit the EMT progression and retinal vascular proliferation, so then slow down the occurrence and development of DR. The specific molecular mechanisms of 25 (OH) D3 have not been revealed yet. Therefore, in order to lay the foundation for the treatment of DR, more in vitro and in vivo experiments are needed for further exploration.

## Conclusion

To sum up, 25 (OH) D3 can effectively alleviate the impaired viability, oxidative stress, and ferroptosis of high glucose-induced hRMVECs, and its mechanism of action may be achieved by down-regulating the expression of miR-93.

## Supplementary Information

**Additional file 1.** Western Blot's original impression.

## Data Availability

The datasets used and/or analyzed during the current study are available from the corresponding author on reasonable request.

## Abbreviations

25 (OH) D3: 25-hydroxyvitamin D3
hRMVECs: Human retinal microvascular endothelial cells
DR: Diabetic retinopathy
ROS: Reactive oxygen species
MDA: Malondialdehyde
GSH: Reduced glutathione
Fe^2+^: Ferrous ion
GPX4: Glutathione peroxidase 4
DM: Diabetes mellitus
PRP: Panretinal photocoagulation
Vit D: Vitamin D
