# Supplementary figures and images for: 25-hydroxyvitamin D3 inhibits oxidative stress and ferroptosis in retinal microvascular endothelial cells induced by high glucose through down-regulation of miR-93
Source: BMC Ophthalmol. 2023 Jan 13;23:22. doi: 10.1186/s12886-022-02762-8 (PMC9840274; doi:10.1186/s12886-022-02762-8)

**Figure 3B**


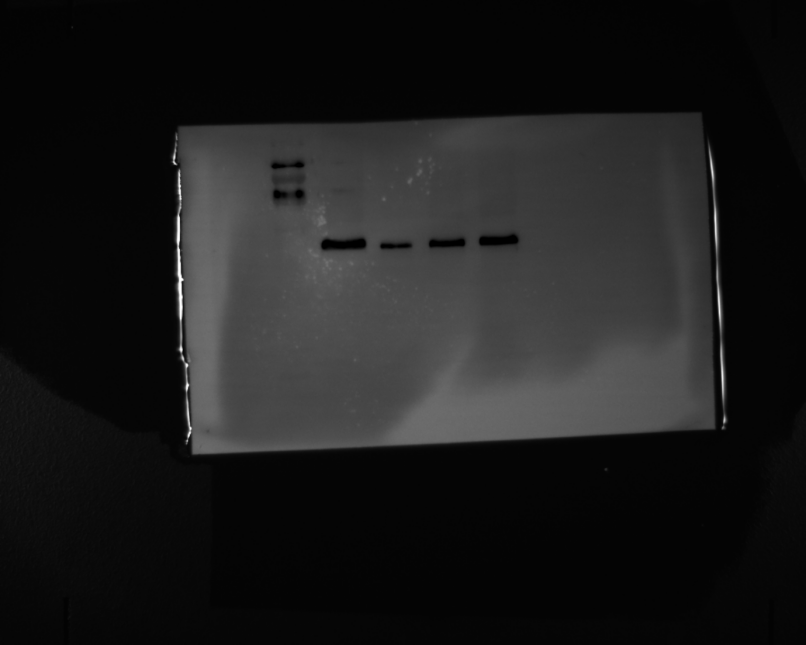


GPX4


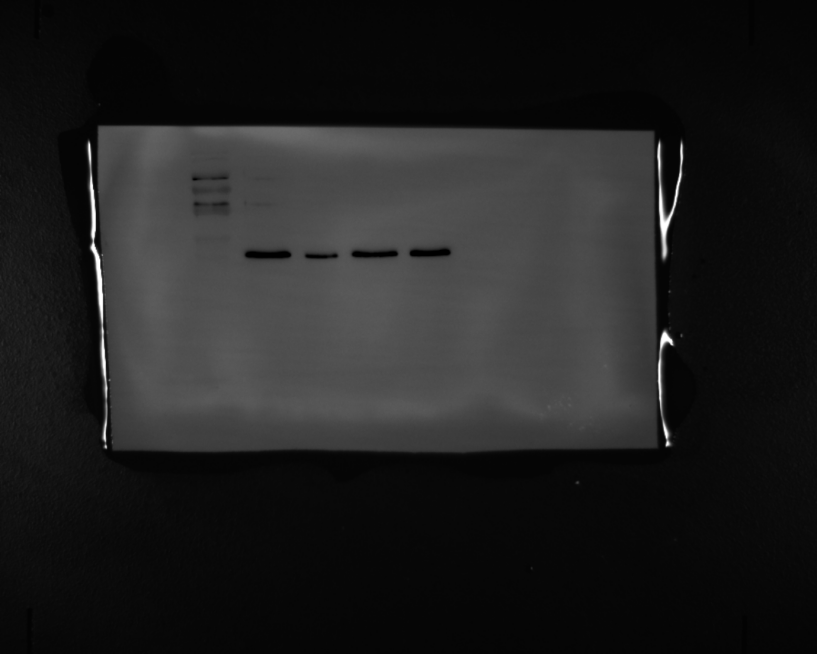


SLC7A11


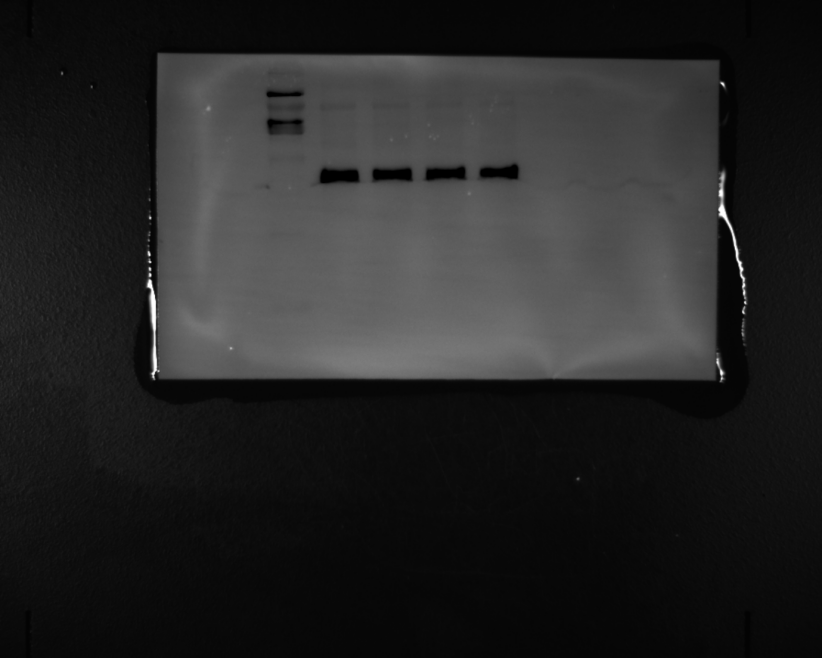


GAPDH

**Figure 5F**


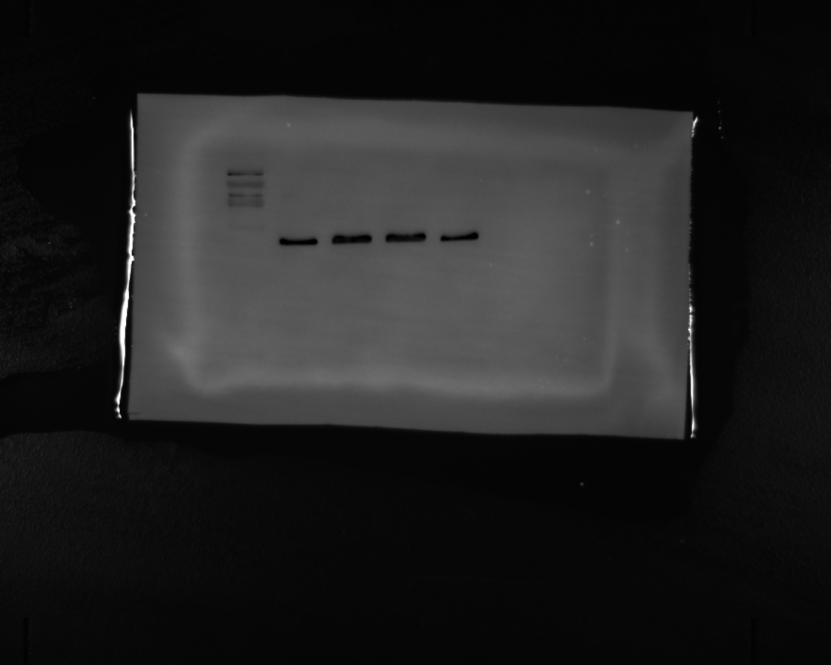


GPX4


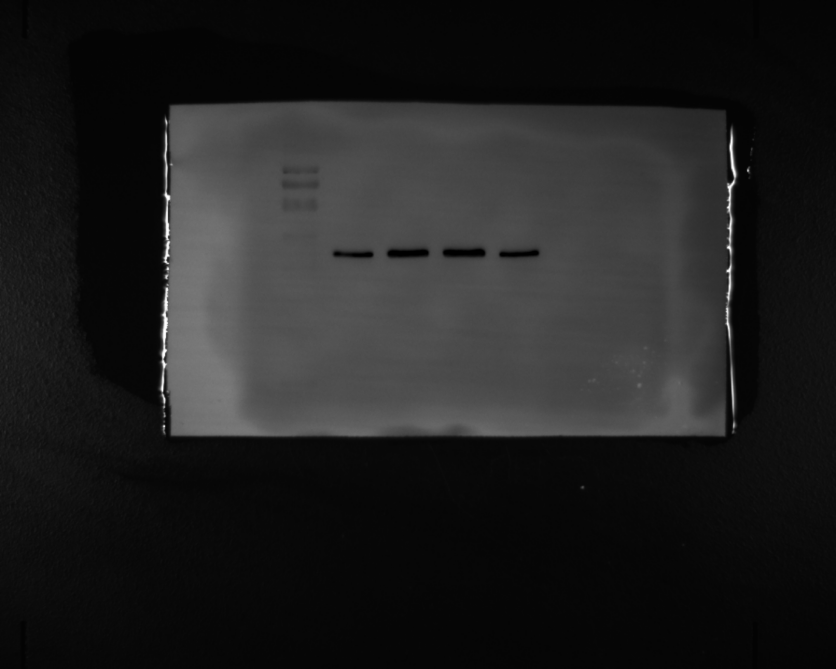


SLC7A11


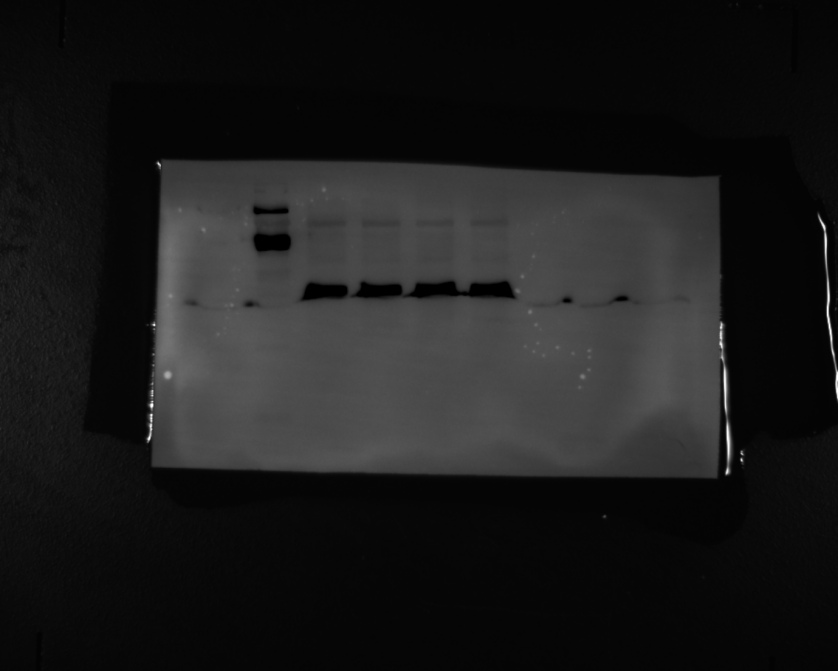


GAPDH

Supplement: Supplementary file 1 — Additional file 1. Western Blot's original impression. [file 12886_2022_2762_MOESM1_ESM.docx]
